# A surrogate FRAX model for Mongolia

**DOI:** 10.1007/s11657-025-01501-y

**Published:** 2025-02-16

**Authors:** M. Jaalkhorol, H. Johansson, S. Avirmed, A. Dashtseren, O. Bruyère, M. Lorentzon, N. C. Harvey, E. V. McCloskey, J. A. Kanis

**Affiliations:** 1 Department of Health Research, Graduate School, Mongolian National University of Medical Sciences, Ulaanbaatar, Mongolia; 2 Centre for Metabolic Bone Diseases, University of Sheffield, Sheffield, UK; 3 Graduate School, Mongolian National University of Medical Sciences, Ulaanbaatar, Mongolia; 4 Department of Preventive Medicine, School of Public Health, Mongolian National University of Medical Sciences, Ulanbaatar, Mongolia; 5 Research Unit in Public Health, Epidemiology and Health Economics, University of Liege, Liege, Belgium; 6 Sahlgrenska Osteoporosis Centre, Institute of Medicine, University of Gothenburg, Gothenburg, Sweden; 7 MRC Lifecourse Epidemiology Centre, University of Southampton, Southampton, UK; 8 NIHR Southampton Biomedical Research Centre, University of Southampton and University Hospital Southampton NHS Foundation Trust, Southampton, UK; 9 Division of Clinical Medicine, School of Medicine and Population Health, Mellanby Centre for Musculoskeletal Research, University of Sheffield, Sheffield, UK

**Keywords:** Mongolia, Hip fracture, FRAX, Surrogate model

## Abstract

**Summary:**

A surrogate FRAX® model for Mongolia has been constructed using age- and sex-specific hip fracture rates for mainland China and age- and sex-specific mortality rates from Mongolia.

**Introduction:**

FRAX models are frequently requested for countries with little or no data on the incidence of hip fracture. In such circumstances, the development of a surrogate FRAX model is recommended based on country-specific mortality data but using fracture data from a country, usually within the region, where fracture rates are considered to be representative of the index country.

**Objective:**

This report describes the development and characteristics of a surrogate FRAX model for Mongolia.

**Methods:**

The FRAX model used the ethnic-specific incidence of hip fracture in mainland China, combined with the death risk for Mongolia in 2015–2019. Intervention thresholds were developed based on fracture probabilities equivalent to women with a prior fragility fracture, and their impact was assessed in a referral cohort comprising men at age 50 and above and postmenopausal women. The number of hip fractures in 2015 and 2050 was estimated based on United Nations’ predicted changes in population demography.

**Results:**

The surrogate model gave similar hip fracture probabilities to estimates from China. Age-dependent intervention thresholds for a major osteoporotic fracture ranged from a 10-year probability of 2.4% at the age of 40 years to 13.7% at the age of 90 years. In the cohort of those eligible for assessment, 46% of men and 36% of women were eligible for treatment because of a prior fracture. Based on intervention thresholds, a further 0.5% of men and 7.0% of women would be eligible for treatment. It was estimated that 440 hip fractures arose in 2015 in individuals aged 50 years and older in Mongolia, with a predicted 4.3-fold increase expected by 2050, when 1896 hip fractures are expected nationally.

**Conclusion:**

The surrogate FRAX model for Mongolia provides an opportunity to determine fracture probability within the Mongolian population and help guide decisions about treatment.

## Introduction

In 2008, the then WHO Collaborating Centre for Metabolic Bone Diseases at the University of Sheffield, UK, launched the FRAX® tool for the calculation of 10-year fracture probabilities in women and men from readily obtained clinical risk factors (CRFs) with or without bone mineral density (BMD) measurements at the femoral neck (http://www.shef.ac.uk/FRAX). The algorithm (FRAX) was based on a series of meta-analyses using primary data from population-based cohorts that examined a list of candidate clinical risk factors for fracture [1, 2]. The output of FRAX comprises the probability of a major osteoporotic fracture (hip, spine, distal forearm or proximal humerus) or hip fracture. This probability is in turn dependent upon the risk of fracture and the competing risk of death, both of which vary from country to country [3]. Ideally, data for age-specific incidences of fracture and death should be available for the construction of country-specific FRAX models, but information on fracture incidence is frequently poor or absent. On a positive note, the availability of FRAX has stimulated studies of fracture incidence that can be used for the generation of new FRAX models; specific examples include Armenia, Belarus, Brazil, Kazakhstan, Mexico, Moldova, Russia, Turkey and Uzbekistan [4].

Where data on hip and other fractures are not available, the International Society for Clinical Densitometry and International Osteoporosis Foundation recommend the development of a surrogate FRAX model to be used until country-specific data are collected and made available. Surrogate models are constructed on age- and sex-specific mortality data from the index country, combined with age-specific and sex-specific rates of fracture derived from a country, usually nearby, where fracture rates are considered to be representative of the index country [5]. Of the 86 countries for which a FRAX model is available, 12 FRAX country-specific models currently use surrogate data on fracture risk (Bangladesh, Brunei, Ethiopia, Georgia, India, Kyrgyzstan, Myanmar, Nepal, Pakistan, Palestine, Sri Lanka and Syria). In the absence of epidemiological data on fracture in Mongolia, the present report describes the development of a surrogate FRAX model.

## Methods

Mongolia is a landlocked country in East Asia, bordered by Russia to the north and China to the south. It covers an area of 1,564,116 km^2^ (603,909 square miles), with a population of 3.5 million, making it the most sparsely populated sovereign state [6]. The population of Mongolia is young with a median age of 26.9 years against a global value of 30.3 years and a median age of 40.3 years in the UK [7].

### Development of surrogate model

Given its border with China, it was decided to base the Mongolian model on the fracture rates of mainland China. As described previously, in the absence of incidence data for other sites of major osteoporotic fracture (MOF; clinical spine, distal forearm and proximal humerus), the hip fracture rates were used to estimate these incidences on the assumption that the ratio of hip fracture incidence to these other FRAX outcomes is the same in the index country as that documented in Sweden, Iceland, Canada, Moldova and elsewhere [8–11]. National mortality rates for Mongolia used data from the United Nations for 2015–2019 [12].

### Patient sample for assessment of model impact

Patients at referral clinics in the Songinokhairkhan district of Ulaanbaatar (*n*=230), the Chingeltei district of Ulaanbaatar city (*n*=126), Dornogovi province (*n*=79), the Khuvsgul province (*n*=153), the Tuv province (*n*=117), the Khentii province (*n*=84) and the Gobi-Altai province (*n*=68) were recruited for documentation of FRAX risk factors. The multiple sources were aimed to derive a referral population representative of the country. Men and women aged 40 years and older giving informed consent were included. Exclusion criteria were inability to walk, not being resident in the province or city, taking bone active medication, Parkinson’s disease, alcohol abuse or unwillingness to participate. The study had the approval of the local Ethics Committee. This cohort was used to evaluate the impact of the model and associated intervention and BMD measurement thresholds described below. The study protocol was approved by the Ethics Committee of the Mongolian National University of Medical Sciences (MNUMS, No.: 2024/3–05).

### Intervention threshold 

In assessing the impact of the FRAX model in the referral cohort, men and women with a prior fracture in adult life were assumed to be eligible for treatment in accordance with most assessment guidance [13]. An intervention threshold in individuals without a prior fracture was set at the age-specific 10-year probability of a major osteoporotic fracture (hip, clinical spine, forearm or humerus) equivalent to women with a prior fragility fracture using the surrogate Mongolian FRAX model. Body mass index was set at 27 kg/m^2^ (close to the mean value of the patient sample). Eligibility for treatment was determined in men of age 50 years or more and in postmenopausal women (491 of the total 857 sample, hereafter termed the impact sample) in accordance with established assessment guidelines [13–15]. Thus, eligibility for treatment comprised those with a prior fracture and those in whom MOF fracture probabilities equalled or exceeded the age-dependent intervention threshold. Importantly, in this cohort, bone mineral density (BMD) was measured in the hand and not the femoral neck, so that FRAX-based fracture probabilities were calculated without the inclusion of BMD

### Assessment thresholds guiding BMD measurement

Assessment thresholds for making recommendations for the measurement of BMD were considered [2]:

A threshold probability below which neither treatment nor a BMD test should be considered (lower assessment threshold).

A threshold probability above which treatment may be recommended irrespective of BMD (upper assessment threshold).

The lower assessment threshold was set to exclude a requirement for BMD testing in women without clinical risk factors, as given in current European guidelines [14, 15]. It was therefore set to the age-specific 10-year probability of a major fracture equivalent to women with no clinical risk factors. An upper threshold was chosen to minimise the probability that a patient, characterised to be at high risk using clinical risk factors alone, would be reclassified to be at low risk with additional information on BMD and vice versa [16]. The upper assessment threshold was set at 1.2 times the intervention threshold.

### Hip fracture

The age- and sex-specific incidence of hip fracture was applied to the population of Mongolia in 2015 to estimate the number of hip fractures nationwide in that year. Additionally, future projections were estimated up to 2050 assuming that the age- and sex-specific incidence remained stable. Population demography was taken from the United Nations using the medium variant for fertility [6].

## Results

**Fig. 1:**
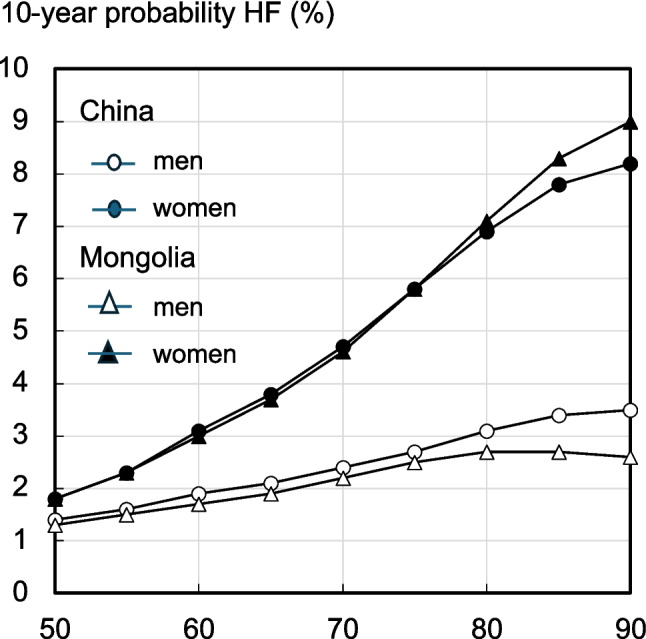
Age-specific 10-year probabilities (%) of a hip fracture (HF) for men or women without clinical risk factors and BMI of 27 kg/m^2^ with unknown BMD, using Mongolian and Chinese FRAX models

The 10-year hip fracture probabilities for Mongolia were similar to those for China at the age of 50 years, but the difference in mean values increased with age, an effect that was more marked for men (Figure 1). With advancing age, the surrogate FRAX model gave lower 10-year fracture probabilities for men and higher probabilities for women at older ages, compared to the model for China, reflecting differences in competing mortality risk. A similar pattern was seen for MOF (data not shown).

Characteristics of the patients studied in the impact cohort are summarised in Table 1. As might be expected in a referral population, there was a high prevalence of a fracture history, parental history of hip fracture, secondary osteoporosis and, in men, current smoking and high intake of alcohol. The distribution of clinical risk factors was similar in the sample of men and women eligible for assessment (in the impact sample, Table 1) though the mean age of men and women was older by 8.5 and 5.4 years, respectively.

**Table 1:** Characteristics of the patients studied

	Whole sample		Impact sample	
	Men	Women	Men	Women
	403	454	204	287
Age (years)—mean (SD)	51.6 (10.4)	53.9 (10.0)	60.1 (7.5)	59.3 (7.8)
BMI (kg/m^2^)—mean (SD)	26.8 (4.6)	27.3 (4.9)	26.5 (4.4)	27.5 (5.0)
Hand BMD T-score—mean (SD)	− 1.81 (1.66)	− 1.92 (1.55)	− 2.28 (1.66)	− 2.39 (1.41)
Previous fracture—*n* (%)	158 (39)	140 (31)	95 (47)	102 (36)
Parental history of hip fracture—*n* (%)	106 (26)	115 (25)	50 (25)	64 (22)
Current smoking—*n* (%)	169 (42)	36 (8)	85 (42)	16 (6)
Glucocorticoid exposure—*n* (%)	20 (5)	27 (6)	11 (5)	13 (5)
Rheumatoid arthritis—*n* (%)	33 (8)	45 (10)	17 (8)	38 (13)
Secondary osteoporosis—*n* (%)	102 (26)	108 (24)	60 (30)	77 (27)
Alcohol 3 or more units daily—*n* (%)	155 (39)	37 (8)	87 (43)	18 (6)
10-year probability MOF (%)—mean (SD)	*N* = 400, 3.1 (2.5)	*N* = 453, 4.6 (3.8)	*N* = 202, 4.0 (2.8)	*N* = 287, 5.6 (4.0)
10-year probability HF (%)—mean (SD)	*N* = 400, 0.8 (1.4)	*N* = 453, 1.1 (1.9)	*N* = 202, 1.3 (1.7)	*N* = 287, 1.5 (2.2)

**Fig. 2:**
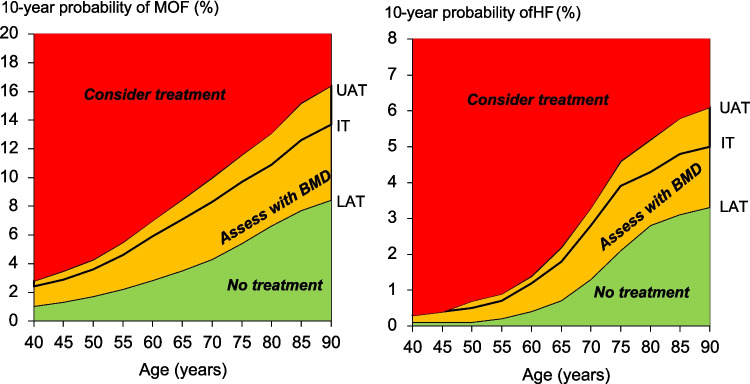
Graphs showing assessment and intervention thresholds in Mongolia for major osteoporotic fracture probability (MOF) and hip fracture probability (HF). The line through the amber area represents the intervention threshold (IT) while the BMD assessment thresholds are shown at the borders of the amber area (upper and lower assessment thresholds; UAT and LAT, respectively)

The intervention threshold in women (set at the age-specific probability of a major osteoporotic fracture equivalent to women with a prior fragility fracture and a BMI of 27 kg/m^2^) rose with age from a 10-year probability of 3.6% at the age of 50 years to 13.7% at the age of 90 years (Table 2 and Fig. 2). Table 2 and Fig. 2 also give the age-specific upper and lower assessment thresholds for recommending the measurement of BMD in the assessment of fracture probability. At the age of 65 years, for example, a BMD test would not be recommended in an individual with a fracture probability below 3.5%. At the same age, a BMD test would be recommended with a fracture probability that lay between 3.5 and 8.5%. Treatment would be recommended without the requirement of a BMD test (for fracture risk assessment, though possibly for monitoring of treatment) in individuals with a fracture probability that exceeded 8.5%. In individuals in whom a BMD test was undertaken, treatment would be recommended in those with a fracture probability that was 7.1% or greater for MOF or 1.8% for hip fracture probability.

**Table 2:** Ten-year probability of a major osteoporotic fracture (MOF) and hip fracture (HF) by age at the intervention threshold (IT), lower and upper assessment thresholds (LAT and UAT) calculated with the Mongolian FRAX model. BMI set to 27 kg/m^2^

Age (years)	MOF			HIP		
	LAT	IT	UAT	LAT	IT	UAT
40	1.02	2.35	2.82	0.05	0.28	0.30
45	1.29	2.89	3.47	0.08	0.35	0.42
50	1.66	3.62	4.34	0.12	0.49	0.59
55	2.16	4.60	5.52	0.22	0.74	0.89
60	2.82	5.86	7.03	0.4	1.16	1.39
65	3.51	7.06	8.47	0.73	1.81	2.17
70	4.33	8.31	9.97	1.31	2.78	3.34
75	5.41	9.70	11.64	2.14	3.87	4.64
80	6.59	10.92	13.10	2.81	4.34	5.21
85	7.67	12.64	15.17	3.14	4.84	5.81
90	8.36	13.69	16.43	3.27	5.04	6.05

The disposition of patients in the eligibility sample is shown in Table 3. Of the 202 men, 46.5% were eligible for treatment, the majority of whom were eligible because of a prior fracture (93 of 94 patients). Had femoral neck BMD facilities been available, densitometry would have been recommended in 21 patients. In the case of women, 42.5% were eligible for treatment. As was the case in men, most women were eligible because of a prior fracture (102 of 122 patients). Had femoral neck BMD facilities been available, densitometry would have been recommended in 116 women. As expected, 10-year probabilities of a MOF were lower in men than in women for all categories.

**Table 3:** Disposition of the eligibility cohort according to 10-year probabilities of a MOF with regard to assessment and intervention thresholds

	Men				Women			
	*n*	%	Probability MOF (%)	BMI (kg/m^2^)	*n*	%	Probability MOF (%)	BMI (kg/m^2^)
All	202	100	4.0	26.5	287	100	5.6	27.5
Prior fracture	93	46.0	6.1	26.4	102	35.5	9.0	27.4
*Without prior fracture*								
Low risk (≤ LAT)	87	43.1	1.9	27.0	57	19.9	2.4	31.0
BMD tests	21	10.4	3.6	24.6	116	40.4	3.9	25.9
High risk (> UAT)	1	0.5	7.2	28.0	12	4.2	7.7	27.0
≤ IT but > LAT	21	10.4	3.6	24.6	108	37.6	3.7	26.0
> IT but ≤ UAT	0	0	-	-	8	2.8	6.2	24.3

*IT*, intervention threshold; *UAT*, upper assessment threshold; *LAT*, lower assessment threshold.

### Fracture projections

Assuming that the fracture rates derived from China were representative for Mongolia, and based on the United Nations estimates of the Mongol population for 2015, we estimated that the annual number of hip fractures in men and women aged 50 years or older in Mongolia in 2015 totalled 440, comprising 146 in men and 294 fractures in women. The number of hip fractures is estimated to increase progressively by calendar year with a 4.3-fold increase to 1896 by 2050 (Table 4).

**Table 4:** Estimated total number of hip fractures (ICD-10 codes S72.0, S72.1, S72.2) in men and in women at age 50 years and older in 2015 projected up to 2050 in Mongolia

	2015	2020	2030	2040	2050
Men	146	180	264	400	539
Women	294	350	553	933	1357
Total	440	530	817	1333	1896
Increase (%)	-	120	186	303	431

## Discussion

This paper describes the development of a surrogate FRAX model for Mongolia, utilising hip fracture rates in mainland China and mortality data from Mongolia. With advancing age, the surrogate model provided marginally different estimates of fracture probability for hip fractures in men and women in Mongolia compared with the Chinese model. The differing probabilities in Mongolia reflect differences in age-specific mortality between the two countries. Importantly, the differences will have little impact on the stratification of risk, since little change in the rank order of fracture probability has been shown in other surrogate models [17–22].

An obvious limitation of this study is the assumption that the fracture rates in Mongolia are similar to those in mainland China—an assumption that cannot be tested. Research is required to derive Mongolian hip fracture incidence data with which to refine this FRAX model.

A further limitation, though one shared with the majority of current FRAX models, is that the model was constructed using incidence data on hip fracture only, rather than all major osteoporotic fractures. The latter are calculated from the hip fracture incidence on the basis that the age- and sex-specific relationship between these fractures and hip fractures is similar to that reported in Malmo, Sweden [8]. Importantly, this commonality of pattern has been observed in other studies where data has allowed its assessment including Canada [9], Iceland [10], the USA [23], the UK [24], Australia [25] and Moldova [11, despite marked differences in incidence between these countries [3]. This commonality of pattern is supported by register studies, which indicate that in those regions where hip fracture rates are high, so too is the risk of forearm fracture and spine fractures (requiring hospital admission) [26, 27].

The impact of assessment algorithms on the population identified for treatment has been determined in several countries most usually in population-based samples [10, 28–38] and, more rarely, referral patients [39–43]. The eligibility cohort in the present study differs in that it was an outpatient referral population but not specifically for skeletal assessment. Notwithstanding, the study identified a high proportion of patients with a prior fragility fracture and a high requirement for treatment. Eligibility for treatment was found in more than 50% of men and women indicating a large unmet need in Mongolia. At present, DXA is not available in Mongolia which impairs somewhat the assessment strategy. However, FRAX without BMD performs similarly to the use of BMD alone [44], and patients identified by FRAX respond to treatment [45, 46] which reinforces the case for treatment assessment based on prior fracture and FRAX without BMD.

In summary, a surrogate FRAX model has been created for Mongolia. The model provides the opportunity to determine fracture probability among the population and help guide decisions about treatment.

## Data Availability

Primary data available upon a reasonable request.
